# Alcohol use among Nigerian university students: prevalence, correlates and frequency of use

**DOI:** 10.1186/s12889-019-7104-7

**Published:** 2019-06-13

**Authors:** Anthony Idowu Ajayi, Eyitayo Omolara Owolabi, Oluyinka Olutola Olajire

**Affiliations:** 1 Department of Sociology, Faculty of Social Science and Humanities, University of Fort Hare, East London, South Africa; 2 Population Dynamics and Reproductive Health Unit, African Population and Health Research Centre, APHRC Campus, Nairobi, Kenya; 3 Department of Nursing Science, Faculty of Health Sciences, University of Fort Hare, East London, South Africa; 4 Department of Behaviour Studies, Redeemer’s University, Ede, Osun State Nigeria

**Keywords:** Alcohol use, University students, Nigeria, North Central, Religiosity, Family support

## Abstract

**Background:**

Globally, alcohol use is responsible for 320 deaths every hour, and the impact is more among those in the younger age group. Despite the adverse health and social challenges associated with alcohol use, alcohol remains the most used and abused psychoactive substance among young adults. Our study aimed at determining the prevalence, correlates and frequency of alcohol use among young adults in two Nigerian universities. We further explored the role of family structure, family support and religion/religiosity on alcohol use in this study setting. Such findings could help to inform public health policy formulation in the country.

**Methods:**

This was a cross-sectional study conducted in two selected universities in the North Central region of Nigeria. The study was conducted among a final sample of 784 students selected using stratified random sampling. An interviewer-administered questionnaire was used to collect data on ever and current alcohol use and frequency of alcohol use between February and April 2018. The data were analysed using descriptive and inferential statistics.

**Results:**

The level of ever and current use of alcohol was 43.5 and 31.1%, respectively. The mean frequency of alcohol use among the study participants was three days, but ten days among current alcohol users. In the adjusted model, male sex, age above 19 years, infrequent attendance of religious rituals, and belonging to rich/middle-class family were significantly associated with a higher likelihood of ever use and current use of alcohol, while living in the same household as one’s father was associated with lower odds of current and ever use of alcohol.

**Conclusion:**

There is a high rate of lifetime and current use of alcohol among university students in the study setting. Alcohol use was significantly associated with living with parents, religion and religiosity. Both high and low socioeconomic status were associated with alcohol use. There is a need to implement measures in controlling alcohol manufacturing and marketing as well as policies regulating alcohol outlets establishment around educational institutions as well as the working hours in such outlets. Finally, there is a need to organise interventions aimed at reducing this unhealthy social norm among students in this setting.

## Background

Alcohol is the most widely used psychoactive substance, and its use remains a significant public health concern [1–4]. Alcohol use is a risk factor for over 60 disease conditions and injuries, including non-communicable diseases [5]. In furtherance, harmful alcohol use is associated with mental and behavioural disorders [4]. In the 2016 Global Burden of Disease Study, alcohol use was reported to be the seventh leading risk factor for disability and premature mortality and the foremost risk factor for risk-attributable disease burden among people aged 15 to 49 years [5]. Globally, an estimated 2.8 million deaths (which represent 320 deaths every hour) were recorded as a result of hazardous alcohol use. An additional 1.6 and 6.0% of the Disability-adjusted Life Years among females and males is associated with alcohol use [5, 6].

Although the highest rate of harmful alcohol use is often found in the developed economies, African countries are increasingly demonstrating a drift towards this unhealthy lifestyle behaviour [7, 8]. Alcohol forms part of many social gatherings in Africa, and it is central to many cultural activities [9–11]. Also, alcohol sale is a source of income for many in Africa, and as a result, little attention is paid to it both by the government and the populace, neglecting the detrimental health and social effects [7]. The burgeoning issue of alcohol use in Africa is also compounded by the ease of availability, heterogeneity of its production, increase in unlicensed liquor outlets and lack of restrictions on alcohol advertisements, which points to the absence of effective alcohol control policies [8, 12–14]. Even in countries where alcohol control policies are already enacted, both within and outside Africa, there is still often a poor level of coordination, and direct and indirect advertisements of alcohol persist with little regulation [6, 15].

Sub-Saharan African countries, including Nigeria, are confronted with a high prevalence of alcohol use. Evidence from the 2016 Global burden of disease study estimated that Nigeria was one of the countries with the highest prevalence of current alcohol use among adults 15 years and older in sub-Saharan Africa (SSA), 40 to 59.9% at a population level, for both males and females [5]. Some scholars have ascribed the absence of a working policy on alcohol in Nigeria to the sabotaging efforts of the alcohol manufacturing companies on the formulation of effective alcohol control policies [12, 15].

Alcohol use is considered the main risky behaviour among adolescents, young adults and students in general [15–19]. Harmful alcohol use among this cohort is strongly associated with various health and mental disorders such as suicidal ideation, aggressiveness, self-harm and alcohol dependency [19–21]. Likewise, harmful use of alcohol can lead to functional impairment among students, which may result in poor academic performance, as well as increased drop-out rates [22–25]. There is also an associated increase in risky sexual behaviour among students who use alcohol in a harmful way [26–28]. Alcohol abuse among this group is often accompanied by the use of other psychoactive substances, which further increase the negative effects on individuals [29, 30]. In addition, alcohol use among this sect could also have an economic impact such as its negative effect on labour market outcomes, specifically, labour market earnings and attachment [31].

There are several underlying drivers of this social behaviour among students. In addition to socio-demographic factors such as age, gender and socio-economic status of students [3, 16, 32], some scholars have posited that the change in lifestyle behaviour that accompanies tertiary education, as well as the perceived freedom from parental control or supports further heighten this risky behaviour [15, 21, 33]. Other scholars have found an association between harmful alcohol use among students and dysfunctional family settings, as well as the influence of media adverts [6, 19, 34]. Another author highlighted the protective effect of religion on alcohol use [35]. Various other reasons have been purported to contribute to harmful alcohol use among this age group, and these include peer pressure, academic-related stress and workload and unhealthy competitions among peers [21, 36, 37]. Pertaining to the frequency of alcohol use, gender play a significant role and males are more likely to engage in binge drinking more frequently than their female counterparts [38]. There is a fairly substantial evidence base for justifying the implementation of prompt and effective measures for curtailing harmful alcohol use among university students in Nigeria.

Although alcohol use studies have been conducted among high school and university students in Nigeria [1, 17, 29, 31, 33, 39], most of these studies have been confined to a particular region of the country, mostly the South-Western Region, with few studies conducted in the Northern parts [1, 39], where the rate of alcohol use is assumed to be higher. Our study aimed to determine the prevalence of alcohol consumption among university students in the North Central region and to identify risk and protective factors associated with alcohol consumption. Specifically, we explored the influence of religion, frequency of religious attendance, as well as social and family-related factors on ever use, current use and frequency of alcohol use. The findings from this study will provide a picture of patterns of alcohol use and identify factors contributing to harmful alcohol use among university students in Nigeria. This information will be useful in informing public health policies, and the development of the strategies and interventions to reduce harms among Nigeria’s student population.

## Methods

### Study design

This study was conducted in two universities in the North Central region of Nigeria between February and April 2018. The data used for this research was part of a larger study that examined sexual risk behaviour and sexual health and wellbeing of adolescents and young adults in Nigerian Universities. The detailed methodology has been published elsewhere [40]. The study was conducted at the University of Ilorin in Kwara state and Nasarawa state Univerisity in Nasarawa state. There are an estimated 45,000 students in the two universities. Only male and female students from these universities are eligible for the study. Overseas university students were excluded from the study. Using the Cochrane formula for estimating sample size for a descriptive cross-sectional study, at a confidence level of 95%, a precision level of +/− 5%, a sample size of 400 participants is required for each university, after adjusting for possible missing responses. Overall, 400 males and 400 females were included in the study; however, only 784 participants returned completed questionnaires. Participants were selected using stratified sampling. Students were stratified to sex and year of study to ensure representativeness. Participants were recruited randomly from all the faculties in the university at the lecture venues during general courses in each faculty. All the recruited participants were interviewed in private office space provided by a staff of the university.

### Data collection

The questionnaire used in this study was self-designed and pre-piloted before administering to study participants. Prior to data collection, a pilot study was conducted among 20 participants in a university in the South West region of the country. The feedback from the pilot study was used to modify the questionnaire. Research assistants, recruited and trained specially for this study, administered the instrument to consenting students.

The main outcome variable of the study is alcohol use, which was operationalised as ever used alcohol and last month use of alcohol. Frequency of alcohol use was measured by asking participants to state the number of days they drank alcohol over the last month.

There are three main sets of explanatory variables, which are demographic factors (such as age and sex), religious background and religiosity, and family factors (such as family structure, family support, father alive, live with father, mother alive, live with mother and perceived family social status). Age was categorised into 15–19 years, 20–24 years and 25 years and over, while sex was grouped into male and female. The religious background was grouped into Christianity and Islamic religion. Frequency of religious attendance was grouped into frequently (attend religious activities at least twice a week), moderately frequent (attend at least one religious activity in a week), and infrequent (attend a religious event once in a month or a year). Family structure was grouped into four, which are nuclear family, single parent family, polygamous family and foster family. Participants were asked to rate the level of support they get from home as adequate, moderate, insufficient or no support. Family support may be interpreted as both financial and social support because both are inter-related. Participants were also asked whether they perceive themselves as coming from a wealthy, middle class or low-income family.

### Ethical consideration

The ethical review committee of the University of Fort Hare, South Africa (Reference number: GONO11) and Ondo State Health Research Ethics Committee, Ondo State, Nigeria (Reference number: NHREC/18/08/2016), approved the study protocol. Written consent was obtained from all participants. For the few participants (*n* = 55) included in this study who were aged 17 years at the time of the study, assent and parental consent were obtained before their participation. We explained the study objectives and use of information obtained to all participants. Also, participants were told that they were free to skip any question they were not comfortable answering or stop the interview at any time. Anonymity, confidentiality, and privacy were ensured throughout the study.

### Data analysis

The data analysis was performed using the IBM Statistical Package for Social Sciences. The variables of interests were subjected to descriptive and inferential statistics. Simple frequency distribution, percentage, mean and standard deviation were used to describe the outcome variables. We also performed normality test on the variable, ‘frequency of alcohol use’. To examine the correlates of ever use and current use of alcohol, Pearson Chi-square Statistics were estimated. Also, a bivariate binary logistics regression was estimated to examine the independent effect of each explanatory variable on ever use and current use of alcohol. Finally, a multivariate analysis was performed to examine significant predictors of ever use and current use of alcohol. The analysis was performed at a 95% confidence level, and an Alpha value less than 0.05 was considered statistically significant. Sampling weight was assigned for all analysis given the sampling method adopted.

## Results

The mean age of study participants was 21.80 (SD = 3.37) years. The result of the weighted and unweighted demographic characteristics of the study participants are presented in Table 1. Most participants were below the age of 25 years (66.9%), Christians (73.4%), attended religious gathering frequently (64.4%), lives in the same household as their father (83.4) and mother (89.5%), receives adequate support from home (70.9%), from a nuclear family (58.2%) and middle income family (81.8%).

**Table 1:** Weighted and unweighted socio-demographic characteristics of study participants

Variables	Unweighted frequencies and percentages		Weighted frequencies and percentages	
	n	%	n	%
Sex				
Male	402	51.3	390	49.8
Female	382	48.7	390	50.2
Age				
17–19	219	27.9	212	27.0
20 and above	565	62.1	572	73.0
Religious Background				
Christian	574	73.4	573	73.2
Islam	208	26.6	210	26.8
Frequency of religious rituals attendance				
Frequently	512	65.3	512	65.4
Infrequently	272	34.7	271	34.6
Father alive				
Yes	654	83.4	654	83.5
No	130	16.6	129	16.5
Live in the same household as your father				
Yes	567	72.3	555	70.8
No	217	27.7	229	29.2
Mother alive				
Yes	701	89.5	701	89.6
No	82	10.5	82	10.4
Live in the same household as your mother				
Yes	642	81.9	637	81.5
No	142	18.1	145	18.5
Living arrangement				
Single parent	199	25.5	198	25.3
Nuclear	454	58.2	455	58.4
Polygamous	90	11.5	90	11.5
Foster parents	37	4.7	37	4.8
Family support				
Adequate	555	70.9	556	71.0
Moderate	167	21.3	166	21.2
Insufficient	44	5.6	44	5.6
No support at all	17	2.2	17	2.2
Perceived parent’s social economic status				
Wealthy family	100	12.8	101	12.9
Middle income family	640	81.8	639	81.7
Low income family	42	5.4	42	5.4

### Alcohol use among university students

The level of ever and current use of alcohol was 43.5 and 31.1% respectively. However, the proportion varies by age, sex, religious affiliation, father alive, living in the same household as father and mother, family support, family structure and perceived socio-economic status of the parents (Table 2).

**Table 2:** Adjusted and unadjusted model showing independent predictors of ever use of alcohol

Variables	Ever used alcohol	Never used alcohol	Unadjusted Odds ratios (CI)	Adjusted Odds ratios (CI)
All	341 (43.5)	442 (56.5)		
Sex				
Male	199 (51.0)	191 (49.0)	1.84 (1.38–2.45)***	1.59 (1.16–2.17)**
Female	142 (36.1)	251 (63.9)	1	1
Age				
Above 20 years	270 (47.4)	300 (52.6)	1.80 (1.30–2.51)***	1.68 (1.16–2.44)*
17–19 years	71 (33.2)	143 (66.8)	1	1
Religious Background				
Christian	263 (45.9)	310 (54.1)	1.45 (1.05–2.00)*	1.64 (1.14–2.36)*
Islam	78 (37.1)	132 (62.9)	1	1
Frequency of religious rituals attendance				
Infrequently	157 (57.9)	114 (42.1)	2.46 (1.82–3.33)***	2.34 (1.69–3.25)***
Frequently	184 (35.9)	328 (64.1)	1	1
Father alive				
Yes	267 (43.4)	348 (56.6)	0.98 (0.69–1.38)	2.33 (1.15–4.74)*
No	74 (43.8)	95 (56.2)	1	1
Live in the same household as your father				
Yes	224 (40.4)	331 (59.6)	0.65 (0.47–0.88)**	0.42 (0.22–0.81)**
No	117 (51.1)	112 (48.9)	1	1
Mother alive				
Yes	296 (42.3)	403 (57.7)	0.64 (0.41–1.01)	1.29 (0.64–2.65)
No	45 (53.6)	39 (46.4)	1	1
Live in the same household as your mother				
Yes	257 (40.3)	381 (59.7)	0.49 (0.34–0.71)***	0.69 (0.38–1.24)
No	84 (57.5)	60 (42.3)	1	1
Family structure				
Nuclear	176 (38.9)	279 (61.3)	0.62 (0.47–0.83)**	0.74 (0.51–1.08)
Polygamous/single-parent/foster parent	165 (50.3)	163 (49.7)	1	1
Family support				
Adequate support	219 (39.5)	336 (60.5)	0.56 (0.41–0.76)***	0.76 (0.54–1.07)
Inadequate support	123 (53.7)	106 (46.3)	1	1
Perceived parent’s social economic status				
Rich	55 (54.5)	46 (45.5)	1.67 (1.09–2.54)*	1.93 (1.22–3.05)**
Poor/middle income	286 (41.9)	397 (58.1)	1	1
School Location				
Nasarawa	154 (38.5)	246 (61.5)	0.66 (0.49–0.87)**	0.75 (0.53–1.05)
Ilorin	187 (48.8)	196 (51.2)	1	1

***p-value < 0.001, **P-value < 0.005, *P-value< 0.05, *CI* confidence interval

Adjusted and unadjusted logistic regression models were used to examine the key determinants of lifetime use of alcohol. Four sets of covariate variables were included in the regression analysis based on our review of literature on determinants of alcohol use; demographic, religion, family factors (living with father and mother, family structure, family support, parents social status) and geographical covariates (university location). The unadjusted model is the baseline, which was used to assess the net effect of each explanatory variable on lifetime use of alcohol. In the unadjusted model, male sex, age above 19 years, Christianity, infrequent attendance of religious fellowships, and having a rich parent were associated with a higher likelihood of ever use of alcohol. While living with one’s father, living with one’s mother, nuclear family structure, adequate family support, and studying in Nasarawa state university were all associated with lower odds of ever use of alcohol.

In the adjusted model, male sex, age over 19 years, Christianity, infrequently attending religious rituals and having a rich parent remain significantly associated with higher odds of ever use of alcohol. The magnitude and direction of effects remained even after including other control variables. However, only living in the same household as one’s father was associated with reduced odds of ever used alcohol. Even though family support, school location, family structure and living with mother were significantly associated with ever use of alcohol in the unadjusted model, the effect size reduced and no longer reach the significant level. However, the direction of the relationship remains.

As shown in Table 3, the level of current alcohol use was 31.1%; however, the proportion varies by sex, age, frequency of ritual attendance, living in the same household as parents, living arrangement, family support and perception of parent’s socio-economic status.

**Table 3:** Adjusted and unadjusted model showing independent predictors of current use of alcohol

Variables	Current alcohol use		Unadjusted Odds ratios (CI)	Adjusted Odds ratios (CI)
	Yes	No		
All	243 (31.1)	540 (68.9)		
Sex				
Male	152 (37.8)	250 (62.2)	1.71 (1.26–2.33)**	1.46 (1.04–2.05)*
Female	97 (25.4)	285 (74.6)	1	1
Age				
20 and above	198 (35.0)	367 (65.0)	1.87 (1.29–2.69)**	1.91 (1.26–2.89)*
15–19	51 (23.3)	168 (76.7)	1	1
Religious Background				
Christian	183 (31.9)	391 (68.1)	1.05 (0.74–1.47)	1.23 (0.84–1.82)
Islam	64 (30.8)	144 (69.2)	1	1
Frequency of religious rituals attendance				
Infrequently	120 (44.3)	151 (55.7)	2.39 (1.75–3.27)***	2.15 (1.53–3.02)***
Frequently	129 (25.2)	383 (74.8)	1	1
Father alive				
Yes	202 (30.9)	452 (69.1)	1.25 (0.86–1.82)	2.49 (1.25–4.95)*
No	47 (36.2)	83 (63.8)	1	1
Live in the same household as your father				
Yes	165 (29.1)	402 (70.9)	0.73 (0.53–1.01	0.50 (0.27–0.92)*
No	84 (38.7)	133 (61.3)	1	1
Mother alive				
Yes	212 (30.3)	488 (69.7)	0.57 (0.36–0.91)*	1.25 (0.62–2.55)
No	36 (43.9)	46 (56.1)	1	1
Live in the same household as your mother				
Yes	177 (27.6)	465 (72.4)	0.42 (0.29–0.60)***	0.58 (0.32–1.05)
No	72 (50.7)	70 (49.3)	1	1
Family structure				
Nuclear	127 (28.0)	327 (72.0)	0.66 (0.49–0.89)*	0.78 (0.53–1.16)
Single-parent/ Polygamous/ Foster parents	120 (36.8)	206 (63.2)	1	1
Family support				
Adequate	155 (27.9)	400 (72.1)	0.53 (0.39–0.74)***	0.72 (0.50–1.04)
Inadequate	93 (40.8)	135 (59.2)	1	1
Perceived parent’s social economic status				
Rich	45 (45.0)	55 (55.0)	1.97 (1.29–3.02)**	2.18 (1.36–3.48)**
Not rich	203 (29.8)	479 (90.2)	1	1
School location				
Nasarawa	93 (23.3)	307 (76.8)	0.47 (0.35–0.64)***	0.56 (0.39–0.81)**
Ilorin	150 (39.2)	233 (60.8)	1	1

***p-value < 0.001, **P-value < 0.005, *P-value< 0.05, *CI* confidence interval

Adjusted and unadjusted weighted logistic regression models were used to examine the main determinants of current alcohol use. Four sets of covariate variables were included in the regression analysis based on our review of literature on determinants of alcohol use; demographic, religion, family factors (living with father and mother, family structure, family support, parents social status) and geographical covariates (university location). The results of the unadjusted model show that male sex, age over 19 years, infrequent attendance of religious fellowship and having a rich parent were associated with a higher likelihood of current use of alcohol, while mother alive, living with mother, nuclear family, adequate family support, and schooling in Nasarawa state were significantly associated with lower odds of current alcohol use.

In the adjusted model, male sex, age above 20 years, Christianity, infrequent religious rituals attendance, father alive and having a rich parent remain significantly associated with a higher likelihood of current alcohol use. The magnitude of the effect size and the direction of effect remain. However, only living in the same household as one’s father and studying in Nasarawa state university were associated with reduced odds of current alcohol use. Adequate family support, mother alive, living with mother maintains the negative direction of relationship but no longer reach the level of significance.

### Frequency of alcohol use

The mean frequency of alcohol use among the study participants was three days, but ten days among current alcohol users. The median duration of alcohol use among current users was nine days. We conducted bivariate analyses to select variables for inclusion in the adjusted Ordinary Least Squares (OLS) regression modelling. As shown in Table 4, sex, age, frequency of religious attendance, living in the same household as father and mother, family support and parents’ socioeconomic support were significantly associated with alcohol use frequency. However, family structure was not significantly associated with alcohol use frequency.

**Table 4:** Results of adjusted ordinary least squares regression showing determinants of alcohol use frequency

	Unstandardized coefficient (B)	Standard error	Standardized coefficients (beta)	*P*
Constant	−6.50	2.2		0.004
Sex	−1.24	0.43	−0.10	0.004
Age	0.21	0.07	0.11	0.002
Frequency of religious attendance	1.51	0.22	0.23	0.000
Live as in the same household as your father	1.89	0.61	0.11	0.002
Live as in the same household as your mother	2.02	0.75	0.09	0.007
Parents’ socioeconomic status	−1.70	0.53	−0.11	0.001
Family support	1.45	0.33	0.16	0.000
Living arrangement	0.16	0.29	0.02	0.572
Model adjusted R square	0.16			

## Discussion

Given the recent study published in Lancet, which asserts that no amount of alcohol use is beneficial to the body, and also the associated health, academic, physical and psychosocial effects of alcohol use [19, 21, 26–28], its use among university students is a serious cause for concern. We examined the use of alcohol among adolescents and young adults by surveying two Nigerian universities. Our results show that close to half (43.6%) of the participants had ever consumed alcohol and about one in every three students had consumed alcohol in the last month. The rate of ever use of alcohol in this study is, however, lower than the previously reported rates among other Nigerian students which ranged from 56.5 to 72% while current use of alcohol is within the previously reported range, 27.3 to 33.3% [1, 17, 29, 32]. The plausible reason for this is that many of the referenced studies were conducted in the southern region of Nigeria, which is dominated by Christians. The current study was conducted in the North Central region of Nigeria, which has a large Muslim population. As shown in this study, the use of alcohol is lower among Muslims compared to Christians. Islam forbids alcohol use, and the enforcement of non-use of alcohol among Muslims is more pronounced in the northern region compared to the southern part of Nigeria.

Students who were 20 years and above had a higher prevalence of ever and current use of alcohol. This is not surprising given the legal age of alcohol use in most countries, including Nigeria, is 18 years, and thus the reason for the observed lower prevalence of alcohol among those below 20 years [6]. The World Health Organisation also documented alcohol as the leading risk factor for morbidity and mortality among those aged 20 to 39 years [4]. This indicates that this age group engage more in alcohol consumption than those below this age bracket. Age 20 signifies the transition period between adolescence and adulthood, and it is a time when many young adults are experimenting and trying new things, including consumption of alcohol. In addition to this, this age group are subjected to so much societal pressure, stress and quest to attain success, which may make them embrace the use of alcohol 12,18,19]. Many of the students in this age bracket are also still somewhat dependent for their livelihood, and mostly with many sources of income, so, one might assume they have a little more purchasing power for alcohol, which in a way contribute to alcohol use [41, 42]. Alcohol consumption at an early age and among the younger age groups is a risk factor for alcohol dependency in the future, and such youths are more likely to consume alcohol at a hazardous rate when they grow up [43, 44]. As already known, alcohol users do not only constitute a threat to themselves but also their families, society and their immediate environment. Also, alcohol use among university students is usually associated with poor academic performance, mental and psychological disorders, self-harm and injuries with several other health risks, even though our study did not confirm this [22–25, 38].

Public health interventions aimed at reducing the adverse impacts of alcohol use among students is required, most notably among this age group. Such policies could be geared towards reducing students’ access to alcohol by restricting the establishment of alcohol outlets around school areas and limiting the operating hours of such outlets around the vicinity of the school. Also, given that alcohol price appears affordable for students, increasing the alcohol sale tax is a policy option available to control its use among young people. The effects of alcohol taxation policy on alcohol use and other relevant outcomes are well documented [45, 46].

We found a significant association between male sex and both ever and current alcohol use. Also, male students had a higher frequency of alcohol use. This result is not different from previously documented findings [1, 24, 29, 47]. Males are believed to be more adventurous, and alcohol use among them seems more socially acceptable than for females [16] and hence, the possible higher rate of alcohol use found among them. Likewise, women are seen as social guardians, and the rate of alcohol consumption in public by females is lower than that of males [48]. Even at that, studies are already documenting a close up in alcohol consumption among male and female [49, 50], depending on the socio-economic level. Also, in this study, the observed prevalence among females seems not so far from that of the males.

We found a higher prevalence of ever use of alcohol among Christians and the infrequent religious rituals attendees. The link between religiousness and alcohol use has been well established in the literature [51, 52]. Religion is said to have a protective effect on alcohol consumption and riskier pattern of use. Some religions such as Islam forbid the use of alcohol, due to its psychotropic effects, whereas religions such as Christianity and Judaism do not forbid alcohol consumption and may even use it in rituals [53]. Najjar et al. [51] also reported more favourable attitudes towards alcohol use among non-religious individuals and Buddhist, followed by Christians, with Muslims having the least favourable attitude towards alcohol use. A similar explanation could be applied to the higher prevalence of alcohol use recorded among those who infrequently engage in religious rituals. Olashore et al. [3] also documented similar findings among university students in Botswana.

On family-related factors associated with alcohol use, living in the same household as one’s parents and adequate family support were associated with a lower likelihood of alcohol use. Also, those who were either not living with one or two of their parents or had lost one or two of their parents had a higher frequency of alcohol use. However, family structure arrangement, as well as family support, were not statistically associated with alcohol use in the adjusted model as the effect disappeared after adjusting for confounders. No doubt, living in the same household as parents in a way influences a child’s behaviour [54]. The role of parents as an agent of socialisation for children is essential. Parents teach the normative behaviour acceptable in society to children, and the presence of both parents count towards imbibing these norms. The presence of both mother and father is essential because parents are the central pillar of support of a child; they are responsible for the upbringing of the child. The loss of either of one’s parent is consequential and may present an emotional burden on young people that may in part lead them to use alcohol. This is because parents tend to exert some form of control and discipline over a child. Once the parental control is lost, either as a result of death or divorce, the children may have more freedom than necessary to exhibit an, otherwise, deviant behaviour.

Our finding corroborates studies that have shown that the higher the level of support received from the family is protective of alcohol-related harms, and this occurs predominantly through lower consumption patterns of use [35, 55]. Family support may be in the form of social and financial support, but both are interrelated. Adequate family support represents an investment in the life of a child; as such, children who are adequately supported may not want to disappoint their parents by partaking in an, otherwise, deviant behaviour. Another plausible explanation is that the support received from the family could give the child a sense of entitlement, which may lead them to freely express their concerns, thus, reducing their likelihood of engaging in alcohol use to ease stress, although we do not have this data. However, a family which promotes alcohol use might breed children who participate in alcohol use while a family which frowns at that will more likely raise children who detest alcohol [56].

Likewise, students who perceived their parents to be wealthy engaged more in alcohol use than their counterparts who perceived their parents’ socioeconomic status to be either average or poor. This agrees with the findings of Patrick et al. [57] and Humensky [58], whereby alcohol use was found to be associated with high socio-economic status of young adults. Generally, alcohol use is said to be highest among those with high socioeconomic status, though the impact is often higher among those in the low socio-economic class [40, 41]. One can assume that those who perceive their parents to be rich might have more purchasing power or even have better access to media where alcohol is continually marketed without restrictions [59].

Finally, the frequency of alcohol use was also highest among those who perceived their parents to be poor, closely followed by those who perceived their parents to be rich. The association between socioeconomic status either in terms of finances or other social deprivation and alcohol use is often a paradox. The financial state of a family in a way has the possibility of influencing social behaviours, including alcohol use. It has been highlighted that individuals in the high socio-economic class consume alcohol more while those in the lower socio-economic class engage more in risky drinking [60]. As stated in Hall [61]‘s commentary, the higher risky form of drinking found among those within the lower socio-economic group exposes them to more significant health risks, and this further pushes them down the socio-economic gradient. The same effect was observed in a longitudinal study conducted by Katikiredi and colleagues [62]. Both groups consume alcohol at a hazardous rate and constitute a risk to the society, family, other students and the country at large. What is more, a recent study has shown that no amount of alcohol is considered safe for health, which is against the previous belief that moderate use of alcohol is beneficial to health [5]. There is a need for interventions aimed at alcohol control both at the population level and at the higher institutions of learning and targeting all socio-economic levels.

Alcohol use is widely documented among youths worldwide, and the high rate of alcohol use among the youths in Nigeria is not surprising in the absence of a working or effective policy on alcohol use and marketing in the country [13, 19]. Gone are the days when youths rarely engage in alcohol use due to perceived cultural restraints, with the current rate of pressurising and uncensored adverts from alcohol companies, lack of alcohol policies and organisation of various youth programmes by alcohol manufacturing industries, youths now find alcohol use more appealing [6, 19, 34]. It is worse off among young students, who have a perceived sense of freedom from parental control and as a result, feel free to engage in alcohol use [15, 21, 33]. Young student sees the school years as a time to experiment the various habits they see in their environment, and they sometimes engage in alcohol use for social identity, to improve sexual performance or deal with stress accompanying academic activities [21, 63].

### Strengths and limitations

The cross-sectional nature of the study, as well as the use of self-reporting, are apparent limitations of the study. The cross-sectional nature of the study means that the association reported may not be interpreted as causation. Also, self-reporting of alcohol use, especially among Muslim students, may lead to under-reporting of the prevalence of this behaviour given that their religion forbids alcohol consumption. However, we ensured that each participant completed the questionnaire in a private space to ensure they are free from any external influence. Also, they were informed that their responses will be treated with the utmost confidentiality and that there is no way they can be linked with their answers. Also, males may overstate their alcohol use frequency. Likewise, we could only cover few universities in the selected region, as such, the results of the study should be interpreted with caution and might not be generalizable to the entire university students or young adults in the country. We also did not obtain some important information such as the intensity of alcohol use as well as the parental use of alcohol. However, the large sample size and the evaluation of additional determining factors like religion and frequency of religious rituals are obvious strengths of this study.

## Conclusion

Both ever and last month alcohol consumption were associated with male gender, and is consistent with other studies in Nigeria and elsewhere. Family-related factors such as living in the same household as one’s mother and family support as well as socio-economic status, were also significant predictors of alcohol use. Religion and religiosity also had a significant association with both the rate and frequency of alcohol use. There is a need to implement measures to regulate alcohol manufacturing, marketing and availability, in terms of policies and a need to organise interventions aimed at reducing alcohol use among students in this setting.

## Data Availability

Data from this study will be made available by the corresponding author on request.

## Abbreviations

CI: Confidence Interval
OLS: Ordinary Least Squares
SD: Standard Deviation
SSA: Sub-Saharan Africa
